# Supplementary material for: Genome-wide association meta-analysis identifies five loci associated with postpartum hemorrhage
Source: Nat Genet. 2024 Jul 22;56(8):1597–603. doi: 10.1038/s41588-024-01839-y (PMC11319197; doi:10.1038/s41588-024-01839-y)
Supplement: Supplementary file 1 — Supplementary Figs. 1–6. [file 41588_2024_1839_MOESM1_ESM.pdf]

nature genetics

Supplementary information

https://doi.org/10.1038/s41588-024-01839-y

Genome-wide association meta-analysis identifies five loci associated with postpartum hemorrhage

In the format provided by the authors and unedited

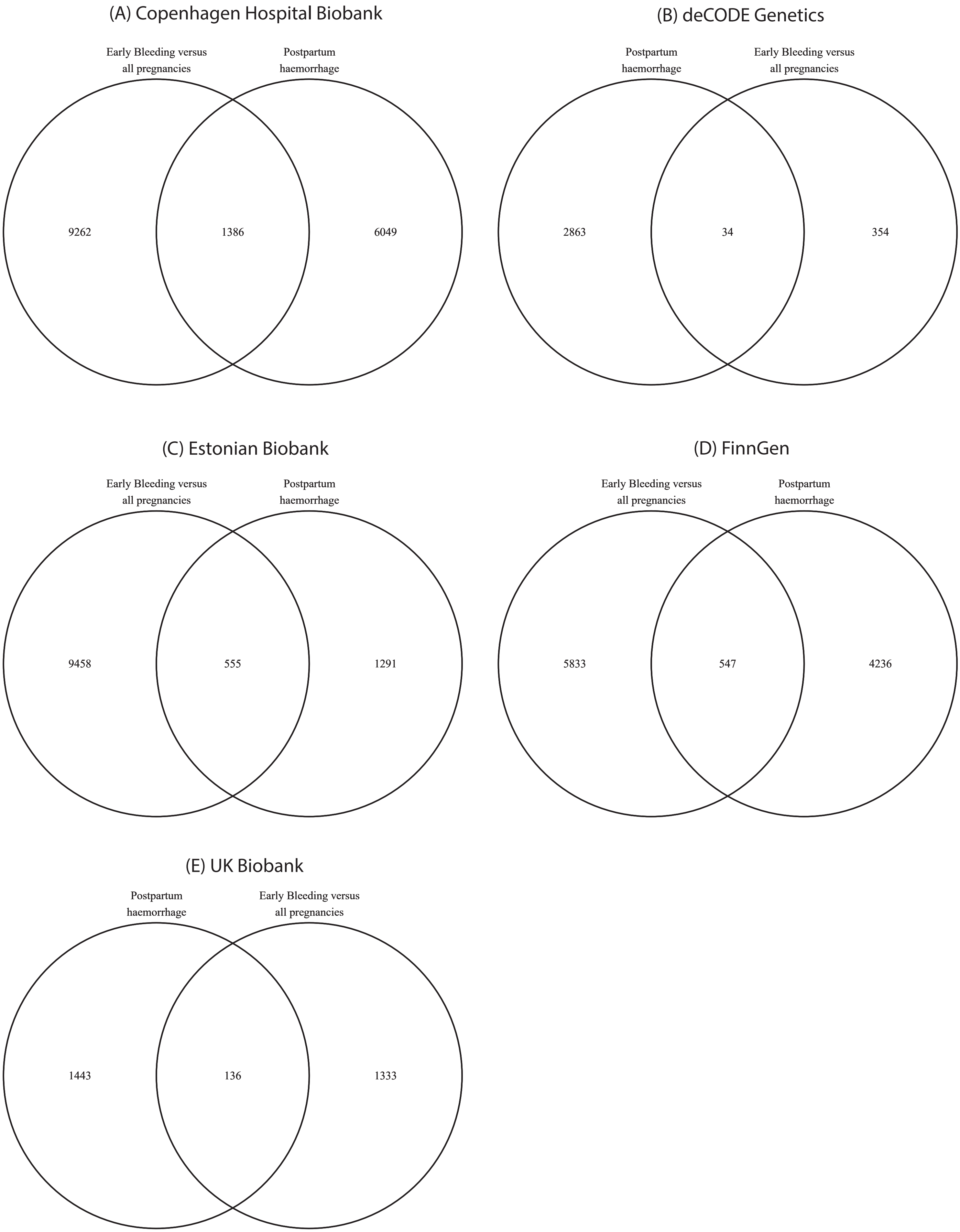

Supplementary Figure 1: Case overlap between early bleeding and postpartum haermorrhage for each cohort.

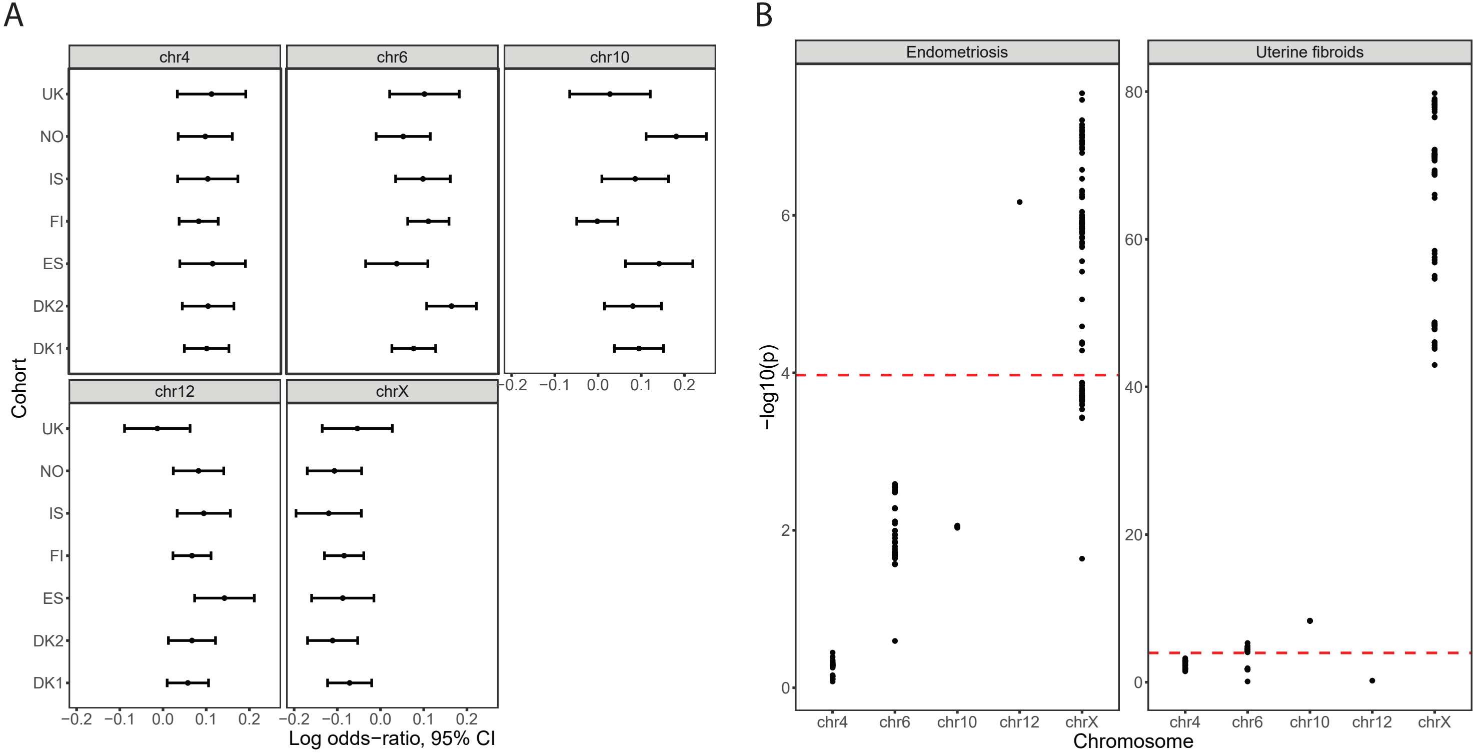

Supplementary Figure 2. (A) Effect sizes in each cohort for the postpartum hemorrhage lead variants, which were largely similar. UK, UK Biobank (England); NO, Norwegian Mother, Father and Child Cohort Study (Norway); IS, deCODE genetics (Iceland); FI, FinnGen (Finland); ES, Estonian Biobank (Estonia); DK2, Copenhagen Hospital Biobank, years 2012-2018 (Denmark), DK1, Copenhagen Hospital Biobank, years 1977-2011 (Denmark). (B) Genome-wide significant variants from the postpartum hemorrhage analysis are also associated with endometriosis and uterine fibroids. The red line indicates the Bonferroni corrected p-value threshold (p < 0.05/(2 loci * 234 variants) = 0.0001).

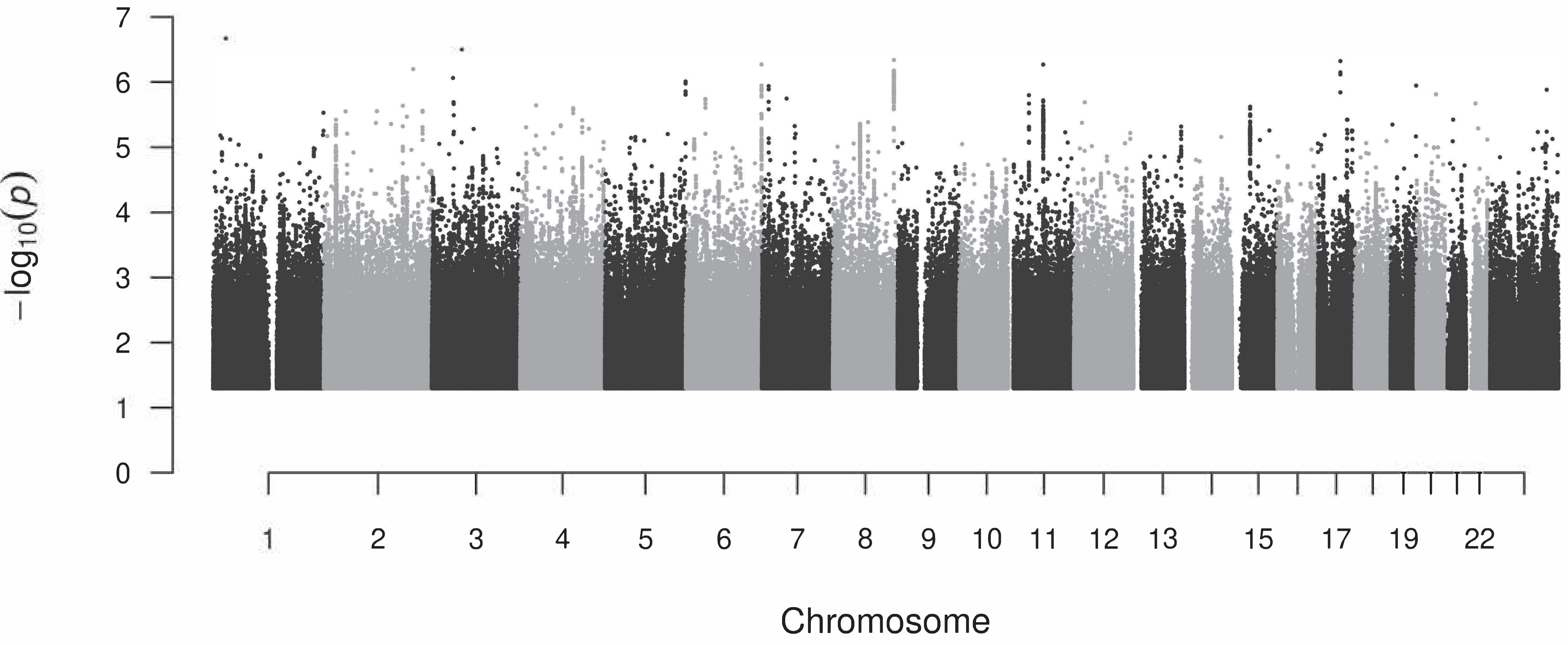

Supplementary Figure 3. Manhattan plot for Early bleeding, all outcomes.

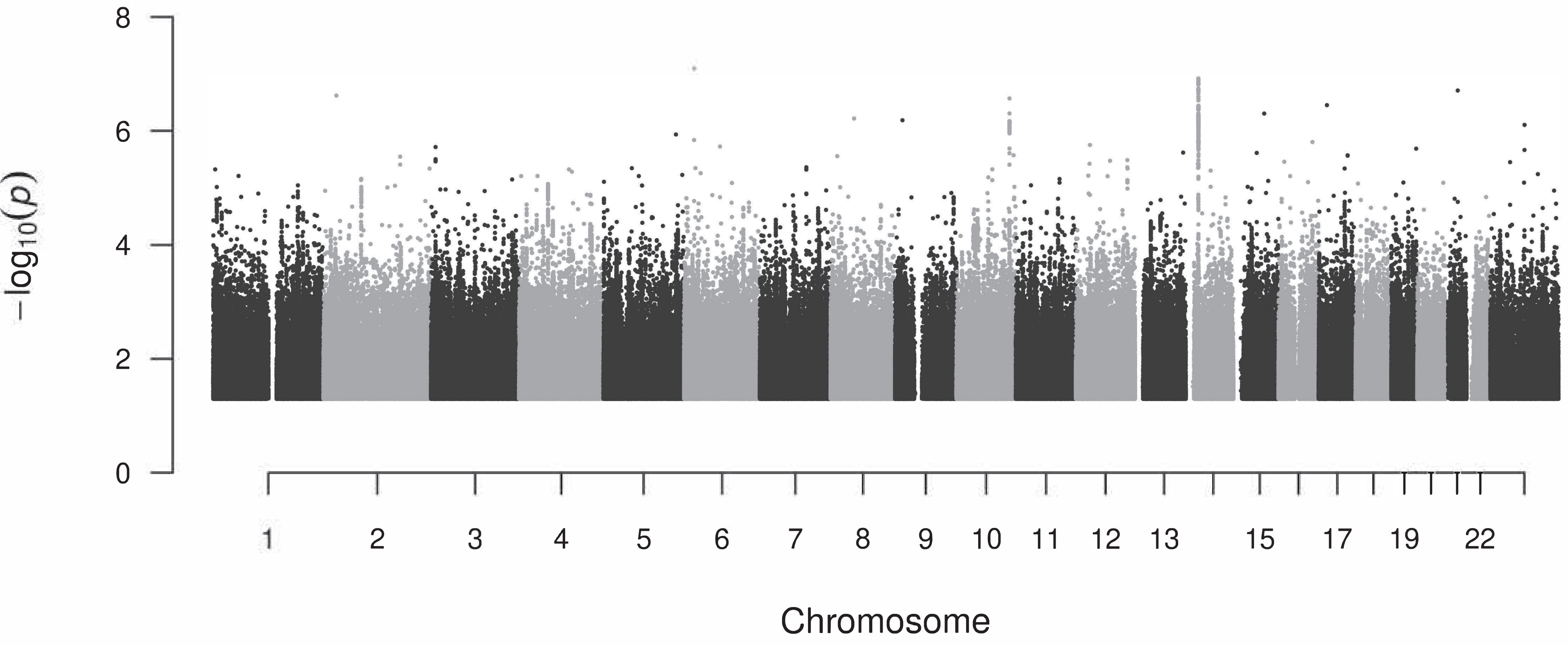

Supplementary Figure 4.Manhattan plot for Early bleeding, ending in live birth.

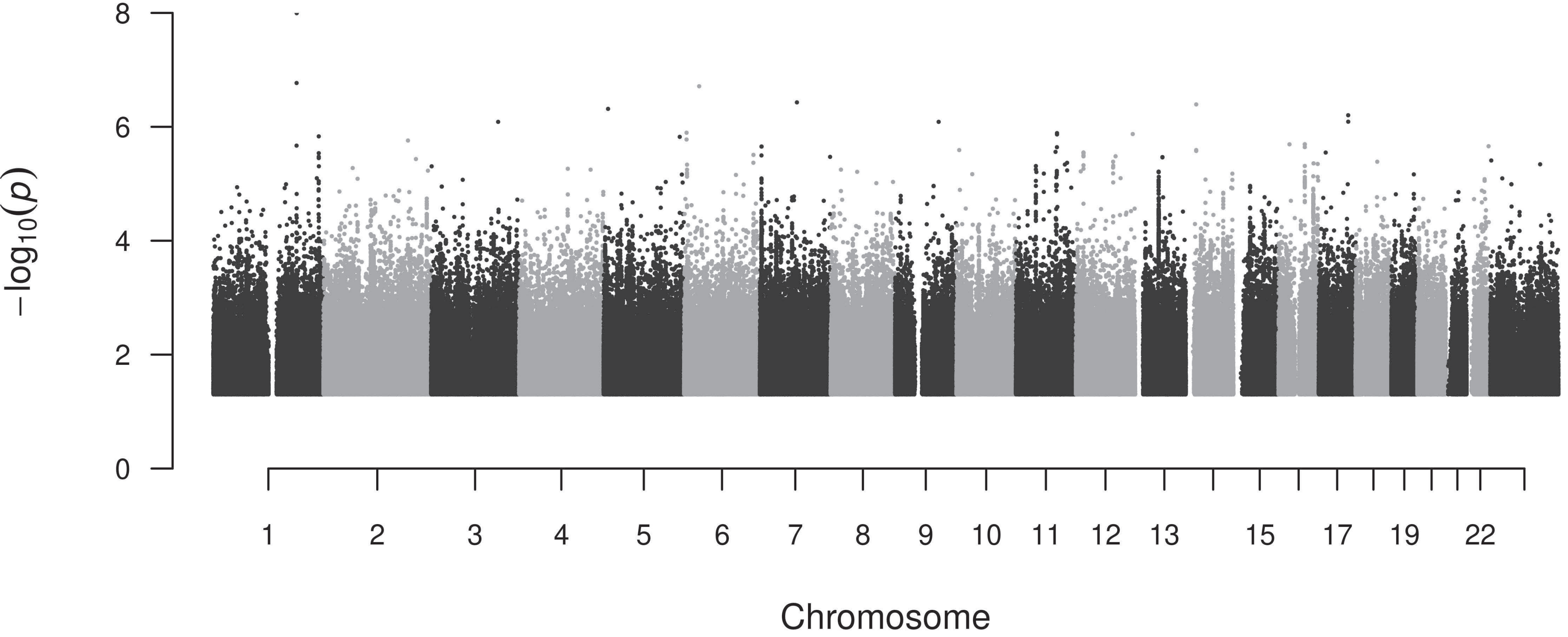

Supplementary Figure 5. Manhattan plot for antepartum bleeding

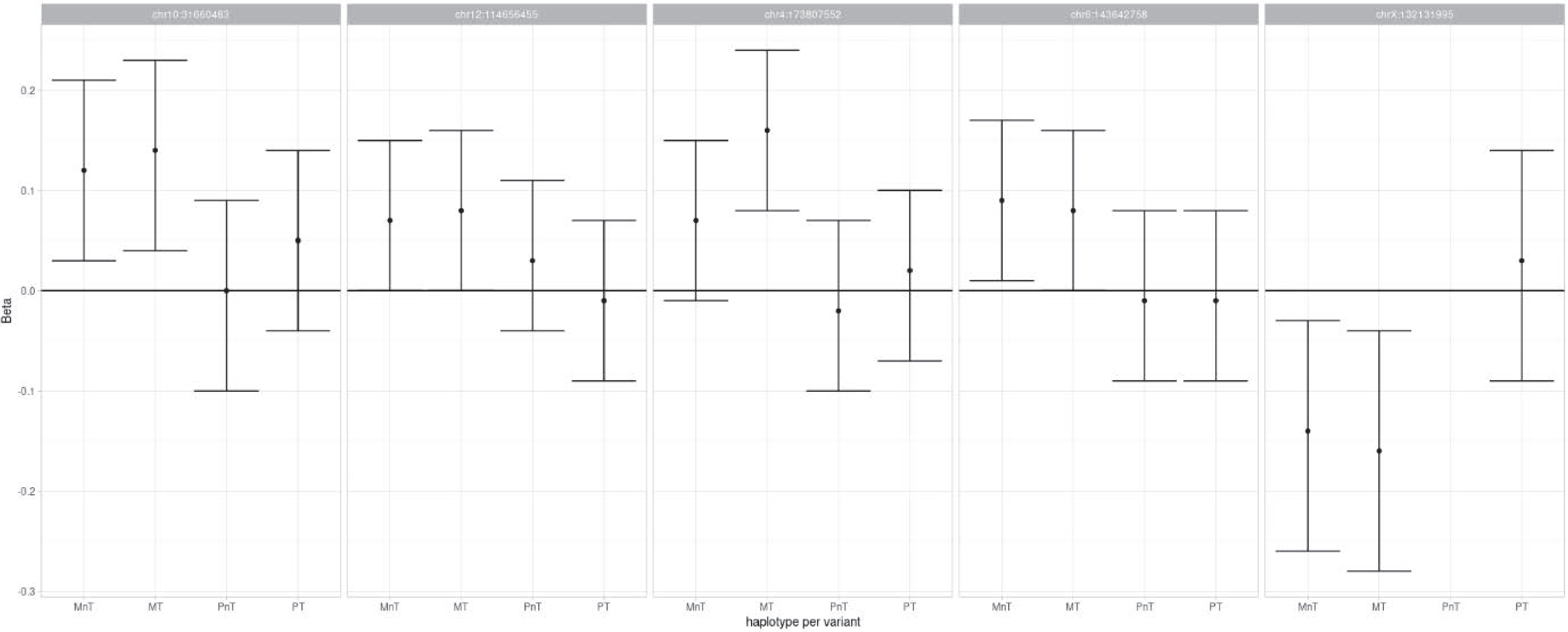

Supplementary Figure 6. Haplotype analysis of the five PPH associated variants in the MoBa and deCODE cohorts. Results suggest that that effect is mediated through the maternal genome. Error bars represent the 95% confidence interval calculated from the Wald statistic. Mnt: maternal non-transmitted; MT: maternal transmitted; PnT: paternal non-transmitted; PT: paternal transmitted.
