# Supplementary material for: Genome-wide association meta-analysis identifies five loci associated with postpartum hemorrhage
Source: Nat Genet. 2024 Jul 22;56(8):1597–603. doi: 10.1038/s41588-024-01839-y (PMC11319197; doi:10.1038/s41588-024-01839-y)
Supplement: Supplementary file 2 — Reporting Summary [file 41588_2024_1839_MOESM2_ESM.pdf]

nature portfolio

Corresponding author(s): Søren Brunak and Henriette Svarre Nielsen

Last updated by author(s): Mar 18, 2024

Reporting Summary

Nature Portfolio wishes to improve the reproducibility of the work that we publish. This form provides structure for consistency and transparency in reporting. For further information on Nature Portfolio policies, see our Editorial Policies and the Editorial Policy Checklist.

Statistics

For all statistical analyses, confirm that the following items are present in the figure legend, table legend, main text, or Methods section.

n/a	Confirmed	
☐	☒	The exact sample size (*n*) for each experimental group/condition, given as a discrete number and unit of measurement
☐	☒	A statement on whether measurements were taken from distinct samples or whether the same sample was measured repeatedly
☐	☒	The statistical test(s) used AND whether they are one- or two-sided<br>*Only common tests should be described solely by name; describe more complex techniques in the Methods section.*
☐	☒	A description of all covariates tested
☐	☒	A description of any assumptions or corrections, such as tests of normality and adjustment for multiple comparisons
☐	☒	A full description of the statistical parameters including central tendency (e.g. means) or other basic estimates (e.g. regression coefficient) AND variation (e.g. standard deviation) or associated estimates of uncertainty (e.g. confidence intervals)
☐	☒	For null hypothesis testing, the test statistic (e.g. *F*, *t*, *r*) with confidence intervals, effect sizes, degrees of freedom and *P* value noted<br>*Give P values as exact values whenever suitable.*
☒	☐	For Bayesian analysis, information on the choice of priors and Markov chain Monte Carlo settings
☒	☐	For hierarchical and complex designs, identification of the appropriate level for tests and full reporting of outcomes
☐	☒	Estimates of effect sizes (e.g. Cohen's *d*, Pearson's *r*), indicating how they were calculated

Our web collection on statistics for biologists contains articles on many of the points above.

Software and code

Policy information about availability of computer code

Data collection	No software was used for data collection
Data analysis	We used publicly available software:<br>GCTA (v1.91.1 beta, https://yanglab.westlake.edu.cn/software/gcta/)<br>ReAct (https://github.com/Paschou-Lab/ReACt, commit 3e285901529628551a078cf99e26a8879714c26d)<br>LDlinkR (v1.3.0, https://github.com/CBIIT/LDlinkR)<br>MAGMA (v1.10, https://ctg.cncr.nl/software/magma)<br>RHE-mc (https://github.com/sriramlab/RHE-mc, commit a3dc6eab08ede92e711ed5532e8aad4708225c95)<br>plink (v1.9, https://www.cog-genomics.org/plink/)<br>LDSCore Regression (https://github.com/bulik/ldsc, commit aa33296abac9569a6422ee6ba7eb4b902422cc74<br>LDPred2 (v1.10, https://github.com/privefl/bigsnpr)<br>ggplot2 (v3.3.3, https://ggplot2.tidyverse.org/)<br>R (v4.3, https://www.r-project.org)

For manuscripts utilizing custom algorithms or software that are central to the research but not yet described in published literature, software must be made available to editors and reviewers. We strongly encourage code deposition in a community repository (e.g. GitHub). See the Nature Portfolio guidelines for submitting code & software for further information.

Data

Policy information about availability of data

All manuscripts must include a data availability statement. This statement should provide the following information, where applicable:

- Accession codes, unique identifiers, or web links for publicly available datasets
- A description of any restrictions on data availability
- For clinical datasets or third party data, please ensure that the statement adheres to our policy

Meta-analysis summary statistics are deposited at https://www. decode.com/summarydata/. FinnGen data are publicly available and were downloaded from https://www.finngen.fi/en/access_results.

URLs for other external data used are as follows: Annotations of candidate cis-regulatory elements, screen.encodeproject.org; EpiMap, compbio.mit.edu/epimap; Remap2022, remap2022.univ-amu.fr; GWAS Catalog, https://www.ebi.ac.uk/gwas/; precomputed LD scores for European populations, https://data.broadinstitute.org/alkesgroup/LDSCORE/eur_w_ld_chr.tar.bz2; Human Protein Atlas, https://www.proteinatlas.org/about/download; NCBI Build 38, https://www.ncbi.nlm.nih.gov/.

Research involving human participants, their data, or biological material

Policy information about studies with human participants or human data. See also policy information about sex, gender (identity/presentation), and sexual orientation and race, ethnicity and racism.

Reporting on sex and gender

The study has focused on the maternal genetics of bleeding in pregnancy, and it must be expected that the mains findings only apply to women. However, we have also used paternal data in the analysis of fetal and maternal transmission.

Reporting on race, ethnicity, or other socially relevant groupings

Please specify the socially constructed or socially relevant categorization variable(s) used in your manuscript and explain why they were used. Please note that such variables should not be used as proxies for other socially constructed/relevant variables (for example, race or ethnicity should not be used as a proxy for socioeconomic status).
Provide clear definitions of the relevant terms used, how they were provided (by the participants/respondents, the researchers, or third parties), and the method(s) used to classify people into the different categories (e.g. self-report, census or administrative data, social media data, etc.)
Please provide details about how you controlled for confounding variables in your analyses.

Population characteristics

All women with at least one pregnancy from each of the participating biobanks were included. A description of the population characteristics is included in the Methods section.

Recruitment

The UK Biobank project is a large-scale prospective cohort study that includes approximately 500,000 individuals from various regions of the United Kingdom .
The Icelandic deCODE Genetics study consists of participants recruited through multiple research projects conducted at deCODE Genetics.
Finngen is a biobank that incorporates both legacy samples, initially collected by the National Institute for Health and Welfare in Finland, and prospective samples acquired from hospital biobanks.
The Copenhagen Hospital Biobank Reproduction Study (CHB-REPRO) is a specialized sub-cohort that focuses on patients with reproductive disorders, drawn from the Copenhagen Hospital Biobank, which itself is based on patient blood samples collected in Danish hospitals.
The Estonian Biobank is a population-based collection that boasts over 200,000 participants, amounting to roughly 20% of the entire Estonian population.
The Norwegian Mother and Child Cohort Study (MoBa) is a population-based pregnancy cohort study executed by the Norwegian Institute of Public Health. The recruitment phase spanned from 1999 to 2008 and included participants from diverse regions of Norway.

Ethics oversight

The deCODE study was approved by the Icelandic National Bioethics Committee (VSN-15-169). The North West Research Ethics Committee reviewed and approved UK Biobank's scientific protocol and operational procedures (REC reference no.: 06/MRE08/65).
Approval of the Copenhagen Hospital Biobank Reproductive Health Study (CHBRHS) was obtained from the Danish National Committee on Health Research Ethics
(NVK-1805807) and the Capital Region Data Protection Agency (P-2019-49).
All study participants provided a signed informed consent, and the study protocol has been approved by the administrative board of the Norwegian Mother, Father and Child Cohort Study, led by the Norwegian Institute of Public Health. The establishment of MoBa and initial data collection was based on a license from the Norwegian Data Protection Agency and approval from The Regional Committee for Medical Research Ethics. The study was approved by the Norwegian Regional Committee for Medical and Health Research Ethics South-East (2015/2425) and by the Swedish Ethical Review Authority (Dnr 2022-03248-01).
Participants in FinnGen provided informed consent for biobank research on basis of the Finnish Biobank Act. Alternatively, separate research cohorts, collected before the Finnish Biobank Act came into effect (in September 2013) and the start of FinnGen (August 2017) were collected on the basis of study-specific consent and later transferred to the Finnish biobanks after approval by Fimea, the National Supervisory

Authority for Welfare and Health. Recruitment protocols followed the biobank protocols approved by Fimea. The Coordinating Ethics Committee of the Hospital District of Helsinki and Uusimaa (HUS) approved the FinnGen study protocol (number HUS/990/2017). The FinnGen study is approved by the Finnish Institute for Health and welfare (approval numberTHL/2031/6.02.00/2017, amendments TH L/1101/5.05.00/2017, THL/341/6.02.00/2018, THL/2222/6.02 .00/2018, THL/283/6.02 .00/2019 and THL/1721/5.05.00/2019), the Digital and Population Data Service Agency (VRK43431/2017-3, VRK/6909/2018-3 and VRK/4415/2019-3), the Social Insurance Institution (KELA) (KELA 58/522/2017, KELA 131/522/2018, KELA 70/522/2019 and KELA 98/522/2019) and Statistics Finland (TK-53-1041-17). The activities of the Est BB are regulated by the Human Genes Research Act, which was adopted in 2000 specifically for the operations of the Est BB. All Estonian Biobank participants have signed a broad informed consent form and analyses were carried out under ethical approval 1.1-12/624 from the Estonian Committee on Bioethics and Human Research (Estonian Ministry of Social Affairs) and data release NOS from the EstBB.

Note that full information on the approval of the study protocol must also be provided in the manuscript.

Field-specific reporting

Please select the one below that is the best fit for your research. If you are not sure, read the appropriate sections before making your selection.

☒ Life sciences ☐ Behavioural & social sciences ☐ Ecological, evolutionary & environmental sciences

For a reference copy of the document with all sections, see nature.com/documents/nr-reporting-summary-flat.pdf

Life sciences study design

All studies must disclose on these points even when the disclosure is negative.

Sample size	GWAS meta analysis. We combined available data from all cohorts.
Data exclusions	The study included all available data except for participants from non-European ethnicities, consistent with the approach taken for all groups.
Replication	The analysis reported here consist of all data from the six different populations, and replication was not performed as the analysis included all data.
Randomization	No randomization was performed. Relevant covariates were included in GWA analysis to account for potential confounding.
Blinding	Group allocation was not relevant to this study, hence blinding was not performed.

Reporting for specific materials, systems and methods

We require information from authors about some types of materials, experimental systems and methods used in many studies. Here, indicate whether each material, system or method listed is relevant to your study. If you are not sure if a list item applies to your research, read the appropriate section before selecting a response.

Materials & experimental systems

n/a	Involved in the study	
☒	☐	Antibodies
☒	☐	Eukaryotic cell lines
☒	☐	Palaeontology and archaeology
☒	☐	Animals and other organisms
☒	☐	Clinical data
☒	☐	Dual use research of concern
☒	☐	Plants

Methods

n/a	Involved in the study	
☒	☐	ChIP-seq
☒	☐	Flow cytometry
☒	☐	MRI-based neuroimaging

Plants

Seed stocks	*Report on the source of all seed stocks or other plant material used. If applicable, state the seed stock centre and catalogue number. If plant specimens were collected from the field, describe the collection location, date and sampling procedures.*
Novel plant genotypes	*Describe the methods by which all novel plant genotypes were produced. This includes those generated by transgenic approaches, gene editing, chemical/radiation-based mutagenesis and hybridization. For transgenic lines, describe the transformation method, the number of independent lines analyzed and the generation upon which experiments were performed. For gene-edited lines, describe the editor used, the endogenous sequence targeted for editing, the targeting guide RNA sequence (if applicable) and how the editor was applied.*
Authentication	*Describe any authentication procedures for each seed stock used or novel genotype generated. Describe any experiments used to assess the effect of a mutation and, where applicable, how potential secondary effects (e.g. second site T-DNA insertions, mosiacism, off-target gene editing) were examined.*
